# Observations on the Use of Coffee as a Cause of Disease

**Published:** 1850-05

**Authors:** Wm. Matthews

**Affiliations:** Nicholsonville, Ind.


### ARTICLE III.

*Observations on the use of Coffee as a Cause of Disease.*—By W. Matthews, M.D., of Nicholsonville, Ind.

Because an article of diet or luxury is in universal use, it is not therefore, necessarily innocuous. On the contrary, several articles which are habitually used by a vast majority of the civilized world, are highly deleterious to the health of the millions who employ them. Most prominent among these are the various preparations of *Alcohol*, *Tobacco*, *Tea*, *and Coffee*.

Passing by the three first named substances, I design, in the observations which follow, calling the attention of the reader to some of the pernicious effects which Coffee, as it is commonly used throughout the Western Country, is capable of exercising upon the constitutions of its consumers, especially of those who use it, as the phrase is, immoderately.—It is true, that persons may live to a great age, and for the most part in the enjoyment of tolerable health, although they swallow daily small doses of poison; yet when the doses are increased, either in frequency or size, a more or less rapid decay and wretchedness is the consequence. Thus, the moderate spirituous drinker, may live to a great age, and even enjoy for the most part, an exemption from pain and sickness; yet no sooner does he become an intemperate drinker, than functional, followed sooner or later, by organic disease, ensues, and life, if not at once destroyed, is rendered burdensome, and the wretched victim drags out a miserable existence of pain and anguish. And the same, I conceive, is true, though in a degree less marked, when coffee is substituted for the alcoholic potations.

Hundreds and thousands of people, especially in the Western States, destroy their health by the intemperate use of coffee, as certainly as the unfortunate inebriate destroys his by the excessive indulgence in his peculiar vice. And it would not seem to be an irrational conclusion, that if large quantities of coffee taken into the stomach prove deleterious to the human system, when taken in less quantities, the same consequences follow, though in an inverse ratio. Admitting that a single cup of coffee, when suitably qualified by cream and sugar, does not act, either upon the nerves of the stomach, or upon the general nervous system, to which its action is chiefly directed, injuriously, yet the validity of the above proposition is not in the least lessened ; for here in the West, where everything is tending to extremes, it is not at all uncommon, indeed it is very common for nervous and exsanguineous females to consume, during a single meal, two, three, four or six cups of strong coffee, almost boiling hot, and perhaps, entirely unqualified by the cream and sugar. Is it not strange, that persons indulging thus intemperately in the use of a pretty active narcotic, should be able to resist its pernicious effects as well as they do? Nor is it uncommon for such individuals to repeat these enormous quantities two or three times daily. Even hot water, thus habitually swallowed, one would suppose could not fail to disorder the stomach, and ultimately lead to troublesome dyspeptic affections ; and such, there can be little doubt, would be the case, especially if accompanied by sedentary habits. Because the gastric juice when greatly diluted, it is well known, loses to a corresponding degree, its solvent power, upon which depends almost entirely the physiology of healthy digestion. But Coffee, when unduly indulged in, undoubtedly interferes with the process of digestion, not only by diluting the gastric juice and scalding the mucous membrane of the primæ viæ but also by disordering, by its narcotic property, the organs of the gastric secretion itself.

Now, it is obvious that the effects of the inordinate use of coffee will be seen, first, in the disordered state of the general nervous system ; and secondly, or concomitantly, in the disorder of the digestive apparatus. From often repeated abnormal stimulation of the nervous system by narcotics, we look for tremors, palpitations, headaches, general debility, and more or less irritability of temper. And such are precisely the symptoms that are evolved in persons addicted to the vicious practice of goading their stomachs, several times daily, with enormous quantities of strong, hot coffee. But most cer tainly, along with the symptoms above enumerated, we have superadded those common to dyspepsia ; such as constipation, a foul tongue, habitually high colored urine, tenderness of the epigastrium, heavy, dull, and persistent pain of, most often, the left hypochondrium, sallow or icteric complexion, lassitude, with a sensation of weariness throughout every part of the body.

For the purpose of more fully illustrating the subject, the two subjoined cases are offered. Similar cases might be mentioned to an indefinite extent, but as little advantage would be likely to result from a repetition of such examples, it is deemed unnecessary to occupy valuable space therewith.

Case I. A boy of twelve years of age, who from infancy had been accustomed to coffee drinking, during the last year had been in a state of ill health. He was pale and exsanguineous; features sharp, and contracted; complained frequently of severe headache; was peevish and ill-tempered. For months he had been suffering from universal tremors; could scarcely raise his hands to his mouth; and indeed, ultimately suffered a paralysis of his right arm and hand. Upon inquiring into the case, it was ascertained, that he was in the habit of consuming three large cups of coffee, once, twice, and not unfrequently three times daily. He took no medicine, but was advised to abandon, *in toto* his long cherished coffee, and use in its stead, a moderate quantity of cow's milk. With the suspension of the coffee, he began to improve, and in a few weeks was in the enjoyment of excellent health.

Now, there can be no question, that if the boy had been allowed to continue his coffee, despite the most judicious medical treatment, he would have remained in ill health, and after years of wretched valetudinarianism, would have become a prey to a pernicious luxury, of the injurious effects of which neither the child nor his parents, ever dreamed.

Case II. W. M., a medical man, of slender and delicate constitution, about thirty years of age, for several years previous to the last one had been the subject of dyspepsia, and its twin brother, hypochondriacism. His bowels were habitually disordered; being either constipated or suffering from Diarrhœa; he was subject to headache; was fretful, emaciated, feeble, short of breath; and upon the slightest exertion, either of body or mind, incipient syncope would be induced; there was irregular action of the heart and arteries—especially of the carotids—which led him into the belief, that he was the subject of incurable cardiac disease. But the most prominent and troublesome symptom was an indescribable persistent pain or uneasiness of the left hypochondrium, which tormented the unfortunate sufferer continually, and from which it was next to impossible to divert his attention, even for a moment. Every imaginary treatment was, of course, adopted in the management of this case; but all to no purpose. The patient being of temperate habits was *dieted* almost to death, yet every symptom seemed to be becoming more grave. The patient was a coffee drinker, and for several years had been in the daily habit of using a single cup, three times daily. This he was finally induced to abandon; and from that day—the day on which he parted with his old and long cherished companion, his improvement has been steady; and at this time his health is sufficiently good to attend to an extensive practice in his profession.

